# The dynamic effects of maternal high-calorie diet on glycolipid metabolism and gut microbiota from weaning to adulthood in offspring mice

**DOI:** 10.3389/fnut.2022.941969

**Published:** 2022-07-28

**Authors:** Jia Zheng, Ling Zhang, Ying Gao, Honghua Wu, Junqing Zhang

**Affiliations:** Department of Endocrinology, Peking University First Hospital, Beijing, China

**Keywords:** maternal high-fat diet, gut microbiota, offspring, glucose metabolism, lipid metabolism

## Abstract

Dysbiosis of gut microbiota can contribute to the progression of diabetes and obesity. Previous studies have shown that maternal high-fat (HF) diet during the perinatal period can alter the microbiota and induce metabolic disorders at weaning. However, whether dysbiosis of gut microbiota and metabolism could be recovered by a normal diet after weaning and the dynamic changes of gut microbiota have not been fully studied. In this study, C57BL/6J female mice were fed with a normal chow (NC) or HF diet for 4 weeks preconception, during gestation, and until pup weaning. After weaning, male offspring were fed with an NC diet until 9 weeks of age. The microbiota of offspring at weaning and 9 weeks of age was collected for 16S rRNA gene amplicon sequencing. We found that dams fed with an HF diet showed glucose intolerance after lactation. Compared with the offspring from NC dams, the offspring from HF dams exhibited a higher body weight, hyperglycemia, glucose intolerance, hyperinsulinemia, hypercholesterolemia, and leptin resistance and lower adiponectin at weaning. Fecal analysis indicated altered microbiota composition between the offspring of the two groups. The decrease in favorable bacteria (such as *norank f Bacteroidales S24-7 group*) and increase in unfavorable bacteria (such as *Lachnoclostridium* and *Desulfovibrio*) were strongly associated with a disturbance of glucose and lipid metabolism. After 6 weeks of normal diet, no difference in body weight, glucose, and lipid profiles was observed between the offspring of the two groups. However, the microbiota composition of offspring in the HF group was still different from that in the NC group, and microbiota diversity was lower in offspring of the HF group. The abundance of *Lactobacillus* was lower in the offspring of the HF group. In conclusion, a maternal HF diet can induce metabolic homeostasis and gut microbiota disturbance in offspring at weaning. Gut microbiota dysbiosis can persist into adulthood in the offspring, which might have a role in the promotion of susceptibility to obesity and diabetes in the later life of the offspring.

## Introduction

Previous studies have demonstrated that an imbalanced diet during early life can increase the susceptibility to obesity and diabetes in the next generations (1). Substantial research and our previous studies both indicated that maternal high-fat (HF) feeding could induce hyperglycemia, glucose intolerance, and insulin intolerance in the offspring at weaning (2–7). Kristin I. Stanford showed that maternal HF diet caused detrimental effects on offspring metabolism at 52 weeks of age (8). However, controversial results were found on the impact of maternal HF diet on adult offspring metabolism when the offspring were delivered a normal diet after weaning. Some research indicated the metabolism disorders in offspring at weaning were recovered by a normal diet in adult offspring (9, 10), whereas other research found that metabolism disorders could persist into adulthood (6, 11). The underlying mechanism of differential impacts of maternal HF diet on adult offspring metabolism is obscure. Growing evidence reveals that modification of offspring microbiome may act as a potential mechanism of development programing (12–14). However, limited information is available on the dynamic changes of gut microbiota and glycolipid metabolism in offspring from weaning to adulthood in offspring mice.

The intestinal microbiome is a unique ecosystem that can influence host energy homeostasis, body adiposity, glucose regulation, insulin sensitivity, inflammation, and hormone secretion (15–17). Disruption of the intestinal microbial ecology is associated with diabetes, prediabetes, and obesity (17). The underlying mechanisms are associated with gut microbiota-derived metabolites, such as lipopolysaccharide (LPS), short-chain fatty acids (SCFA), secondary bile acids, and trimethylamine-N-oxide (TMAO) signaling (18–21). Previous studies have indicated that the gut of offspring can be colonized by microbes as early as the fetal period (12, 22). Babies can obtain mothers’ microbiota mainly from the maternal gut, vagina, and breast milk (23, 24). Thus, the mode of delivery, disease, and maternal diet during the perinatal period can all influence the developing microbiota of infants (23–25). Recent studies have demonstrated that maternal high-calorie diet or gestational diabetes mellitus (GDM) can induce reconfiguration of the microbiome of offspring (26, 27). During gestation or lactation, a maternal HF diet decreased the diversity of gut microbiota and diminished the abundance of non-pathogenic *Campylobacter* in the juvenile gut in primates (27). In human studies, a maternal HF diet induced distinct changes in the neonatal gut microbiome at birth characterized by depletion of *Bacteroides*, which persisted through 4–6 weeks of age (26). Microbiota dysbiosis induced by microbial shifts through GDM mothers caused alteration in neonatal intestinal microbiota and a higher abundance of some viruses in the meconium (12).

Previous studies have reported microbiota dysbiosis and metabolic disorders in offspring at weaning in mice caused by maternal HF diet (6, 28). The offspring shift their diary pattern after weaning. Since the composition of gut microbiota is variable influenced by nutrition, lifestyle, gender, and age (29), the gut microbiota of offspring can be altered by diets after weaning. However, the dynamic changes of gut microbiota and whether the dysbiosis of gut microbiota and metabolism could be recovered by a normal diet after weaning have not been fully studied. Thus, we aimed to investigate the impact of the maternal HF diet on the gut microbiota and glycolipid metabolism at weaning and to further explore whether a normal diet could reverse the dysbiosis of gut microbiota and metabolic disorders caused by a maternal HF diet. In addition, to explore the potential mechanisms underlying the maternal HF diet and metabolic disorders in offspring, the relationship between metabolic parameters and gut microbiota was evaluated in the present study.

## Materials and methods

### Ethics statement

All experimental procedures and protocols were performed according to the Ethics Committee for Animal Experimentation of the Faculty of Peking University First Hospital (No. J201827).

### Animals and diets

For the experiment, 5-week-old female C57BL/6J mice were housed under standard conditions (12-h light/dark cycle; 22 ± 2°C) with *ad libitum* access to food and water. After 1 week of environmental adaptation, all female mice (F0) were randomly fed a normal chow (NC) diet (13% fat; Keao Xieli Feed Co., Ltd., Beijing, China) or a high-fat (HF) diet (60% fat; Keao Xieli Feed Co., Ltd., Beijing, China) for 4 weeks preconception, during gestation, and lactation. The female mice were mated with male mice for 4 days with an NC diet (female:male = 2:1). Pregnancy was confirmed by postcopulatory plugs. The number of pups in each litter was 6–10. To avoid nutrition bias between litters, the litter sizes were culled to 6 for each dam at birth, according to previously published studies (2, 30, 31). All litters were weaned at 3 weeks of age. To avoid confounding causes associated with the estrus cycle and hormone profile of female offspring, we focused on male offspring in this study. Following overnight fasting, one male offspring from every litter was anesthetized with pentobarbital and euthanized. Blood samples were collected using cardiac puncture. Visceral adipose tissue (VAT) and subcutaneous adipose tissue (SAT) were weighed. Fecal samples and blood samples were stored at –80°C for future analysis. The remaining male offspring were delivered on an NC diet until 9 weeks of age. At 9 weeks of age, one male offspring from each litter was anesthetized with pentobarbital and euthanized after 10 h of fasting. Blood samples were collected using cardiac puncture. SAT and VAT were weighed. Fecal samples and blood samples were stored at –80°C for future analysis.

### Glucose tolerance tests in dams and offspring

To avoid stress during gestation and lactation, intraperitoneal glucose tolerance tests (IPGTTs) were carried out in dams when pups were weaned. At weaning age and 9 weeks of age, IPGTTs were carried out in pups after 10 h of fasting. After glucose administration (2 g/kg body weight), blood glucose levels were monitored in the tail vein by using a glucometer and glucose test strips (Contour TS, Bayer, Beijing, China) before (0 min) and at 15, 30, 60, and 120 min. The area under the curve (AUC) of IPGTTs was calculated as previously described (2).

### Serum biochemical parameter measurement

At weaning and 9 weeks of age, blood samples of offspring were centrifuged at 4,000 g for 15 min and stored at –80°C. Enzyme-linked immunosorbent assay (ELISA) kits were used to quantify the concentration of serum insulin (90080; Crystal Chem, Downers Grove, IL, United States), leptin (MOB00B, R&D Systems, Minneapolis, MN, United States) and adiponectin (MRP300; R&D Systems, Minneapolis, MN, United States). Serum total cholesterol (T-CHO), low-density lipoprotein cholesterol (LDL-C), and high-density lipoprotein cholesterol (HDL-C) were detected using commercial kits (A111-1, A112-1-1, A113-1-1, Jiancheng Bioengineering Institute, Nanjing, China). All samples were measured in duplicate.

### Microbial samplings and DNA isolation

Microbial genomic DNA was extracted from cecal contents of male offspring at 3 and 9 weeks of age using a FastDNA Spin Kit for Soil (MP Biomedicals, Santa Ana, CA, United States) following the manufacturer’s protocol. The V3-V4 regions of the bacterial 16S rRNA genes were amplified with primer pairs 338F (5′-ACTCCTACGGGAGGCAGCAG-3′) and 806R (5′-GGACTACHVGGGTWTCTAAT-3′). The PCR product was purified using the AxyPrep DNA Gel Extraction Kit (Axygen Biosciences, Union City, CA, United States) following the manufacturer’s instructions and quantified using a Quantus™ Fluorometer (Promega, United States). Purified amplicons were sequenced on an Illumina MiSeq PE300 platform (Illumina, San Diego, CA, United States).

Raw FASTQ files were de-multiplexed using an in-house perl script, then quality-filtered by Fastp (version 0.19.6) (32), and merged by FLASH (version 1.2.11) (33). The optimized sequences were clustered into operational taxonomic units (OTUs) using UPARSE (version 7.0), with a 97% sequence similarity level (34). The most abundant sequence for each OTU was selected as a representative sequence. Taxonomic information was annotated using the Silva Database (version 128) based on the RDP classifier (version 2.11) (35). Alpha diversity (Simpson index, Shannon index, Chao1, Ace, and Sob) was calculated with mothur (version 1.30.2) (36). The community composition of each sample at different classification levels and beta diversity were determined by QIIME software (version 1.9.1) (37). Differential species (phylum and genera) between the two groups were determined by using the Wilcoxon rank sum test. The influence of the abundance of each species on the differences between groups was evaluated by linear discriminant analysis (LDA) effect size (LEfSe). Phylogenetic Investigation of Communities by Reconstruction of Unobserved States (PICRUSt 2) version 2.2.0 was used to predict metagenome function based on Kyoto Encyclopedia of Genes and Genomes (KEGG) database (38).

### Targeted butyrate quantification

Butyrate concentration in serum of offspring mice was determined and analyzed. A measure of 50 μL serum was added to 100 μL acetonitrile, performed ultrasound at 40 kHz for 30 min at 5^°^C, and then centrifuged at 13,000 g for 15 min at 4^°^C. The supernatant was mixed with 20 μL 3NPH⋅HCL (200 mmol/L) and 20 μL EDC⋅HCL (120 mmol/L), then incubated at 40^°^C for 30 min, and further diluted by acetonitrile (50%, v/v) for detection. The samples were detected using an ExionLC AD system (AB Sciex, MA, United States). Using external calibration curves, butyrate in the samples was quantified.

### Statistical analysis

The data were analyzed using GraphPad Prism 9.0 software. The data were presented as mean ± standard errors (SEM) for normally distributed data. IPGTTs were performed using a two-way ANOVA, followed by Bonferroni’s *post-hoc* test. The difference between the two groups was evaluated using two-tailed Student’s *t*-test. Bioinformatics analysis of the gut microbiota was performed using the Majorbio Cloud platform.^1^ Alpha diversity was assessed using two-tailed Student’s *t*-test. Beta diversity was determined by principal coordinate analysis (PCoA) based on unweighted UniFrac. The Wilcoxon rank sum test was applied to analyze for differential species between the two groups. The correlation between the gut microbiota at the genus level and metabolic parameters was evaluated by using the Spearman correlation coefficient test. A *P*-value less than 0.05 was considered statistically significant.

## Results

### Maternal high-fat feeding negatively impacted glucose metabolism in dams

**FIGURE 1:**
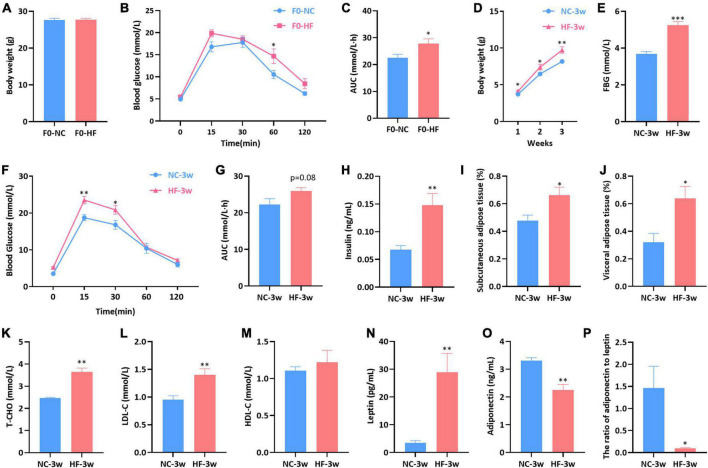
Maternal HF diet impaired glucose metabolism in dams and damaged glycolipid metabolism in offspring at weaning. **(A)** Body weight of dams. **(B)** IPGTT of dams. **(C)** AUC of dams. **(D)** Body weight of offspring. **(E)** FBG of offspring. **(F)** IPGTT of offspring. **(G)** AUC of offspring. **(H)** Serum insulin of offspring. **(I)** Subcutaneous adipose tissue (%) of offspring. **(J)** Visceral adipose tissue (%) of offspring. **(K)** Serum T-CHO of offspring. **(L)** Serum LDL-C of offspring. **(M)** Serum HDL-C of offspring. **(N)** Serum leptin of offspring. **(O)** Serum adiponectin of offspring. **(P)** Ratio of adiponectin to leptin of offspring. Data are represented as mean ± SEM (F0-NC, *n* = 7; F0-HF, *n* = 8; NC-3w, *n* = 5–7; HF-3w, *n* = 6–7). *p < 0.05, ^**^p < 0.01, ^***^p < 0.001 vs. F0-NC, NC-3w. NC, normal chow diet; HF, high-fat diet; IPGTT, intraperitoneal glucose tolerance tests; AUC, the area under the glucose curve; FBG, fasting blood glucose; T-CHO, total cholesterol; LDL-C, low-density lipoprotein cholesterol; HDL-C, high-density lipoprotein cholesterol.

At weaning, no difference in body weight was observed between HF and NC dams (*P* > 0.05, Figure 1A). However, HF-fed dams exhibited glucose intolerance. Compared with NC dams, HF dams exhibited significantly higher glucose levels at 60 min (*P* < 0.05, Figure 1B), as well as a larger AUC value (*P* < 0.05, Figure 1C) during IPGTTs. These results suggested that HF feeding led to glucose metabolic disturbance in dams.

### Maternal high-fat feeding negatively impacted glycolipid metabolism in offspring at weaning

The offspring body weight was assessed weekly from birth to weaning. Compared with the offspring of NC dams, the offspring of HF dams gained more weight in the first (*P* < 0.05), second (*P* < 0.05), and third weeks (*P* < 0.01) (Figure 1D). The offspring displayed increased fasting blood glucose (FBG) induced by the maternal HF diet (*P* < 0.001, Figure 1E). Offspring of HF dams exhibited glucose intolerance at weaning, which presented higher blood glucose levels at 15 min (*P* < 0.01) and 30 min (*P* < 0.05) (Figure 1F), as well as a trend of increased AUC during IPGTTs (*P* = 0.08, Figure 1G). The serum insulin level was higher in the offspring from HF dams than that in offspring from NC dams (*P* < 0.01, Figure 1H).

We further evaluated the impact of the maternal HF diet on lipid metabolism in offspring at weaning. It indicated that SAT (*P* < 0.05, Figure 1I) and WAT (*P* < 0.05, Figure 1J) were both significantly increased in offspring due to the maternal HF diet. Serum levels of T-CHO (*P* < 0.01, Figure 1K) and LDL-C (*P* < 0.01, Figure 1L) were increased in the offspring from HF dams than those from NC dams. However, no difference in the serum HDL-C level was observed between the offspring of the two groups (*P* > 0.05, Figure 1M). The serum leptin concentration was significantly increased in the offspring of HF dams (*P* < 0.01, Figure 1N), and the adiponectin concentration was decreased in the offspring of HF dams (*P* < 0.01, Figure 1O). The ratio of adiponectin to leptin was dramatically decreased in the male offspring of HF dams (*P* < 0.05, Figure 1P).

### Maternal high-fat feeding altered structures and composition of gut microbiota in offspring at weaning

**FIGURE 2:**
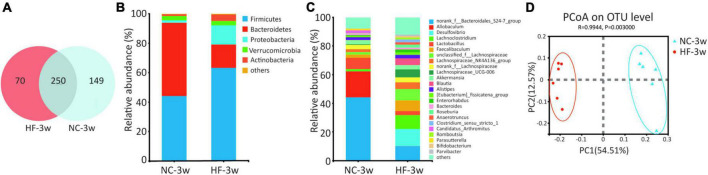
Maternal HF feeding altered structures and composition of gut microbiota in offspring at weaning. **(A)** Venn diagram of the OUTs. **(B)** Relative abundance of the bacterial population at the phylum level. **(C)** Relative abundance of the bacterial population at the genus level. **(D)** PCoA plots of gut communities (NC-3w, *n* = 6; HF-3w, *n* = 6). NC, normal chow diet; HF, high-fat diet. OTUs, operational taxonomic units; PCoA, principal coordinate analysis.

To investigate the effect of the maternal HF diet on gut microbiota in offspring at weaning, we analyzed the microbiota composition of cecal contents using 16S rRNA gene amplicon sequencing and observed distinct differences in microbial ecology between the two groups. Shared and unique germs between the two groups were assessed by analyzing the OTUs. The results indicated 250 shared OTUs, 70 unique OTUs in the offspring from HF dams, and 149 unique OTUs in the offspring from NC dams (Figure 2A). Alpha diversity measures representing richness (Chao1 and Ace) and evenness (Simpson and Shannon) showed a trend of lower diversity in the offspring of HF dams (Table 1). Then, we evaluated the relative abundance of gut microbial composition at the phylum and genus levels in offspring. At the phylum level, Bacteroidetes, Firmicutes, Proteobacteria, Verrucomicrobiota, and Actinobacteria were the most abundant germs (Figure 2B). Bacteroidetes was enriched in the offspring of NC dams, followed by Firmicutes, Verrucomicrobiota, Proteobacteria, and Actinobacteria (Figure 2B), while Firmicutes was enriched in the offspring of HF dams, followed by Bacteroidetes, Proteobacteria, Actinobacteria, and Verrucomicrobiota (Figure 2B). Figure 2C shows the top 25 species at the genus level between the two groups. Beta diversity measures characterizing the differences among groups exhibited that the intestinal microbiota was dramatically separated between the two groups (Figure 2D).

**TABLE 1:** Alpha diversity of gut microbiota in offspring at weaning and 9 weeks of age.

Age	Estimators	NC	HF	*P*-value
**3-week**				
	Ace	275.18 ± 50.818	227.31 ± 30.189	0.075
	Chao 1	288.06 ± 61.556	229.76 ± 28.517	0.062
	Shannon	3.455 ± 0.610	3.578 ± 0.280	0.665
	Simpson	0.1003 ± 0.110	0.0621 ± 0.022	0.424
	Sobs	248.33 ± 46.103	208.67 ± 27.667	0.101
**9-week**				
	Ace	357.23 ± 13.926	321.39 ± 15.809**	0.003
	Chao 1	361.67 ± 16.708	323.73 ± 13.769**	0.003
	Shannon	3.7137 ± 0.287	4.0699 ± 0.403	0.121
	Simpson	0.068455 ± 0.025	0.039476 ± 0.024	0.079
	Sobs	330.5 ± 17.237	300 ± 13.693*	0.011

*p < 0.05, **p < 0.01 vs. NC-9w; NC, normal chow diet; HF, high-fat diet.

**FIGURE 3:**
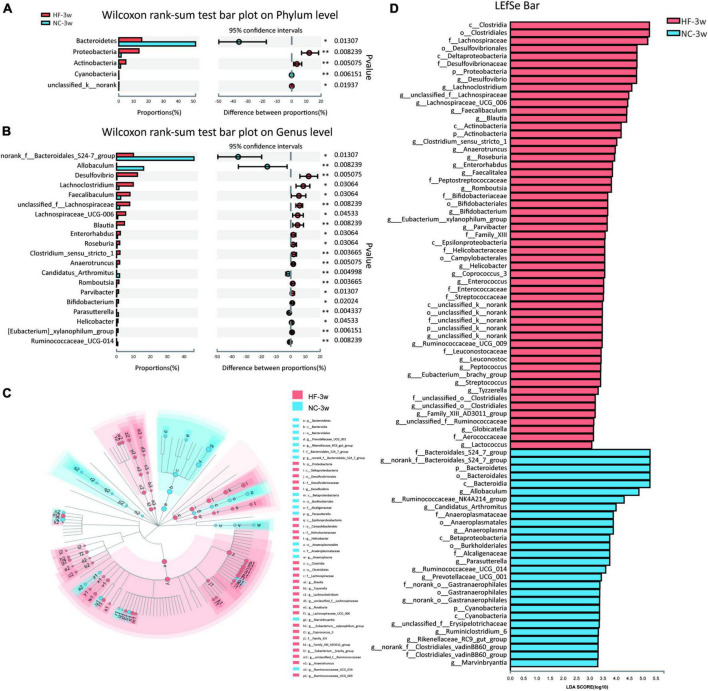
Key bacteria were altered by the maternal HF diet in offspring at weaning. **(A)** Column chart of species abundance at the phylum level. **(B)** Column chart of top 20 species with significant differences at the genus level. **(C,D)** LEfSe analysis of the gut microbiota from the phylum level to the genus level, with LDA values of 2.0 (NC-3w, *n* = 6; HF-3w, *n* = 6). *p < 0.05, ^**^p < 0.01 vs. NC-3w. NC, normal chow diet; HF, high-fat diet. LEfSe, linear discriminant analysis (LDA) effect size.

To further examine alteration in microbiota composition at the phylum and genus levels, the Wilcoxon rank sum test was applied to analyze for differential species between the two groups (Figures 3A,B). Compared to the offspring of NC dams, maternal HF feeding significantly decreased the relative abundance of Bacteroidetes (*P* < 0.05) and enriched the relative abundance of Proteobacteria (*P* < 0.01) and Actinobacteria (*P* < 0.01) at the phylum level (Figure 3A). At the genus level, 43 genera were found to be significantly different between the offspring of the two groups. *Lachnoclostridium* (*P* < 0.05), *Desulfovibrio* (*P* < 0.01), *unclassified f Lachnospiraceae* (*P* < 0.01), Lachnospiraceae UCG-006 (*P* < 0.05), *Faecalibaculum* (*P* < 0.05), *Blautia* (*P* < 0.01), and *Enterorhabdus* (*P* < 0.05) were enriched, whereas *norank f Bacteroidales S24-7 group* (*P* < 0.05) and *Allobaculum* (*P* < 0.01) were decreased in the offspring of HF dams (Figure 3B). To further evaluate the influence of each species on the differences between groups, we analyzed the gut microbiota using LEfSe (Figures 3C,D). The family Bacteroidales S24-7 group, the genus *norank f Bacteroidales S24-7 group* from the family Bacteroidales S24-7 group, and the genus *Allobaculum* and *Ruminococcaceae NK4A214 group* from phylum Firmicutes were the most abundant in the NC group. The family Desulfovibrionaceae, genus *Desulfovibrio* from family Desulfovibrionaceae, and the genus *Lachnoclostridium*, *unclassified f Lachnospiraceae*, *Lachnospiraceae UCG 006*, *Faecalibaculum*, *Blautia*, *Clostridium sensu stricto 1*, and *Anaerotruncus* from phylum Firmicutes were the most abundant in the HF group (Figures 3C,D). Considering the genus *norank f Bacteroidales S24-7 group* is a butyrate-producing bacterium, we further detected the serum butyrate level in offspring at weaning. It indicated that the offspring of HF dams at weaning exhibited a lower serum butyrate level than the offspring of NC dams (Supplementary Figure 1A), which is consistent with the decreased abundance of *norank f Bacteroidales S24-7 group*.

### Correlation analyses between gut microbiota and metabolic parameters in offspring at weaning

**FIGURE 4:**
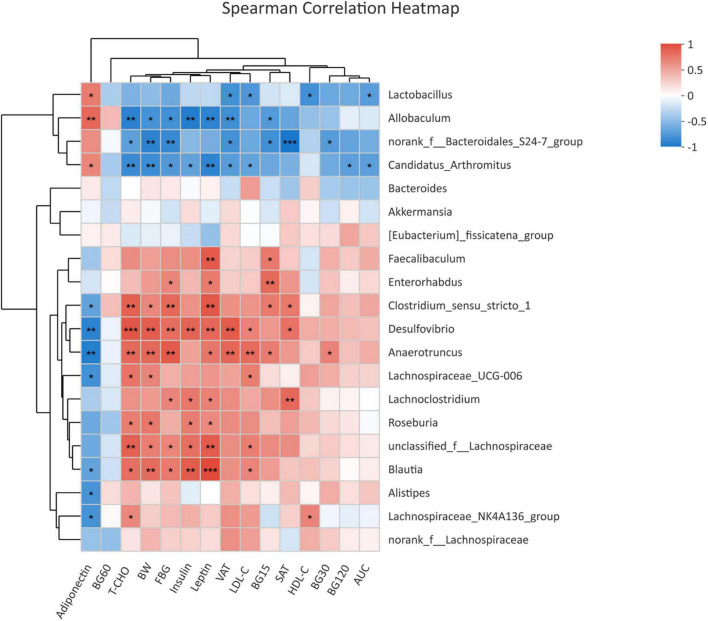
Heatmap of correlation analysis between the differential genera and glycolipid profiles in offspring at weaning. Heatmap of correlation analysis of offspring at weaning (NC-3w, *n* = 6; HF-3w, *n* = 6). *p < 0.05, **p < 0.01, ***p < 0.001 vs. NC-3w. NC, normal chow diet; HF, high-fat diet. BW, body weight; FBG, fasting blood glucose; BG15, blood glucose level at 15 min of IPGTTs; BG30, blood glucose level at 30 min of IPGTTs; BG60, blood glucose level at 60 min of IPGTTs; BG120, blood glucose level at 120 min of IPGTTs; AUC: the area under the glucose curve; T-CHO, total cholesterol; LDL-C, low-density lipoprotein cholesterol; HDL-C, high-density lipoprotein cholesterol; SAT, subcutaneous adipose tissue; VAT, visceral adipose tissue.

To assess the relationship between glycolipid metabolism and gut microbiota, we examined the correlation between metabolic parameters and the abundance of bacterial OTUs at the genus level by the Spearman correlation coefficient test (Figure 4). Genera *Desulfovibrio*, *Blautia*, and *unclassified f Lachnospiraceae*, which were enriched in the offspring of HF dams, were positively correlated with body weight, FBG, leptin, insulin, T-CHO, and LDL-C, among which *Desulfovibrio* and *Blautia* were all negatively correlated with adiponectin. *Enterorhabdus* enriched in HF groups was positively correlated with FBG and leptin. *Lachnoclostridium*, which was abundant in HF groups, was positively correlated with FBG, SAT, insulin, and leptin. The genus *norank f Bacteroidales S24-7 group* and *Allobaculum*, which were decreased in the offspring of HF dams, were negatively correlated with the body weight, FBG, and blood glucose during the IPGTTs, VAT, and T-CHO, among which *Allobaculum* was positively correlated with adiponectin and negatively correlated with insulin and leptin.

### Functional predictions of bacterial communities by Kyoto encyclopedia of genes and genomes pathway database in offspring at weaning

**FIGURE 5:**
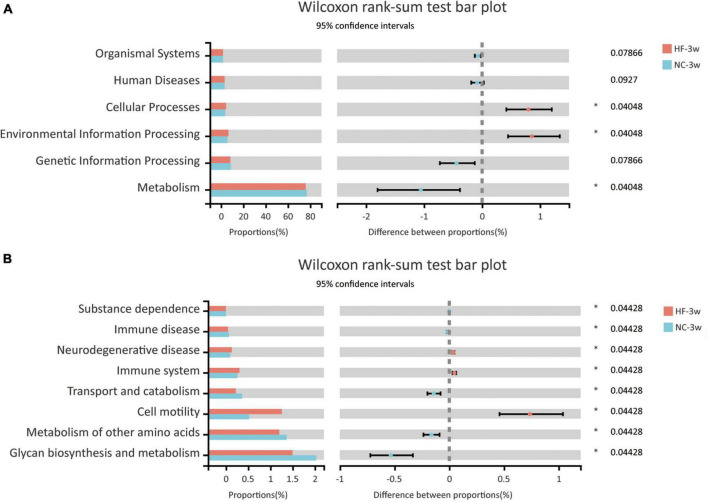
Functional predictions of bacterial communities by the KEGG pathway database in offspring at weaning. **(A)** KEGG pathway level 1. **(B)** KEGG pathway level 2 (NC-3w, *n* = 6; HF-3w, *n* = 6). *p < 0.05 vs. NC-3w. NC, normal chow diet; HF, high-fat diet.

The KEGG pathway analysis was performed to predict functional metagenomic profiles of bacteria in six category pathways (pathway level 1), subcategory pathways (pathway level 2), and secondary pathways (pathway level 3) (39). The level 1 pathway analysis showed that pathways relative to metabolism, cellular processes, and environmental information processing were changed by maternal HF diet, among which the abundance of metabolism-related genes was highest (*P* < 0.05, Figure 5A). The level 2 KEGG pathways indicated that substance dependence, immune disease, neurodegenerative disease, immune system, transport and catabolism, cell motility, glycan biosynthesis and metabolism, and metabolism of other amino acids were altered induced by maternal HF feeding (*P* < 0.05, Figure 5B). In the level 3 KEGG pathway data, the results exhibited significant differences in 51 metabolic functions. Numbers of glucose and lipid metabolism pathways were altered due to the maternal HF diet, such as glycolysis/gluconeogenesis, carbohydrate digestion and absorption, fatty acid biosynthesis, and fatty acid degradation (Supplementary Table 1).

### Glucose and lipid metabolism disorders were recovered by normal chow diet in adult offspring

**FIGURE 6:**
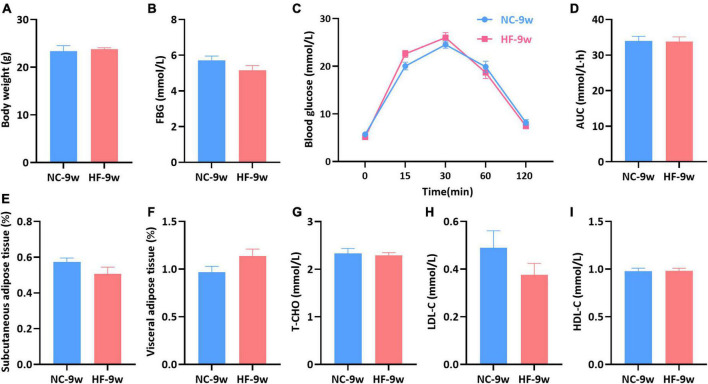
Glycolipid metabolism disturbance induced by the maternal HF diet was recovered in offspring at 9 weeks of age. **(A)** Body weight. **(B)** FBG. **(C)** IPGTT. **(D)** AUC. **(E)** Subcutaneous adipose tissue. **(F)** Visceral adipose tissue. **(G)** Serum T-CHO. **(H)** Serum LDL-C. **(I)** Serum HDL-C. Data are represented as the mean ± SEM (NC-9w, *n* = 8; HF-9w, *n* = 8). NC, normal chow diet; HF, high-fat diet. IPGTT, intraperitoneal glucose tolerance tests; AUC, the area under the glucose curve; FBG, fasting blood glucose; T-CHO, total cholesterol; LDL-C, low-density lipoprotein cholesterol; HDL-C, high-density lipoprotein cholesterol.

To evaluate whether glucose and lipid disturbance could be recovered by a normal diet after weaning, all male offspring were randomized to an NC diet until 9 weeks of age. No significant difference was observed in the body weight, FBG, and glucose tolerance between the offspring of both groups (*P* > 0.05, Figures 6A–D). Meanwhile, no significant difference in the fat mass (SAT and VAT) and lipid levels (T-CHO, HDL-C, LDL-C) was observed between the offspring of both groups (*P* > 0.05, Figures 6E–I).

### Dysbiosis of gut microbiota induced by maternal high-fat diet was partly recovered in adult offspring

**FIGURE 7:**
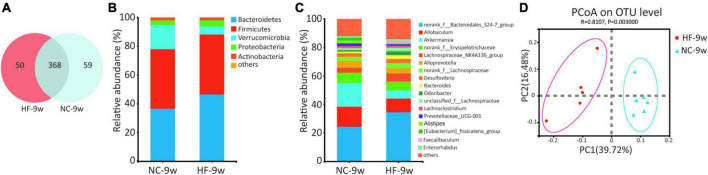
Dysbiosis of gut microbiota was partly restored by a normal diet in offspring at 9 weeks of age. **(A)** Venn diagram of the OUTs. **(B)** Relative abundance of the bacterial population at the phylum level. **(C)** Relative abundance of the bacterial population at the genus level. **(D)** PCoA plots of gut communities (NC-9w, *n* = 6; HF-9w, *n* = 5). NC, normal chow diet; HF, high-fat diet. OTUs, operational taxonomic units; PCoA, principal coordinate analysis.

A maternal HF diet could lead to alteration in structures and composition of gut microbiota at weaning. To evaluate the impact of the NC diet on gut microbiota in offspring after weaning, the microbiota composition of adult offspring was analyzed. Venn diagram indicated 50 unique OTUs in the HF group and 59 unique OTUs in the NC group, with 368 OTUs shared (Figure 7A). Compared with the offspring at weaning, more shared OTUs and fewer unique OTUs were observed in the adult offspring after a normal diet (Figures 2A, 7A). The Ace, Chao, and sobs showed decreased alpha diversity in the offspring of HF dams (Table 1). At the phylum level, Firmicutes was abundant in the offspring of NC dams, followed by Bacteroidetes, Verrucomicrobiota, Proteobacteria, and Actinobacteria, while Bacteroidetes was abundant in the offspring of HF dams, followed by Firmicutes, Verrucomicrobiota, Proteobacteria, and Actinobacteria (Figure 7B). Figure 7C shows differential species at the genus level between the two groups. PCoA on unweighted UniFrac distances exhibited distinct clusters of microbiota composition separated between the two groups at 9 weeks of age (Figure 7D). To evaluate the dynamic changes in beta diversity induced by the NC diet after weaning, we performed PCoA among the offspring of four groups. The results revealed that four distinct clusters of microbiota composition separated among the offspring of four groups. Meanwhile, a 6-week NC diet after weaning partly reversed the effect of gut microbiota induced by the maternal HF diet (Supplementary Figure 1B).

**FIGURE 8:**
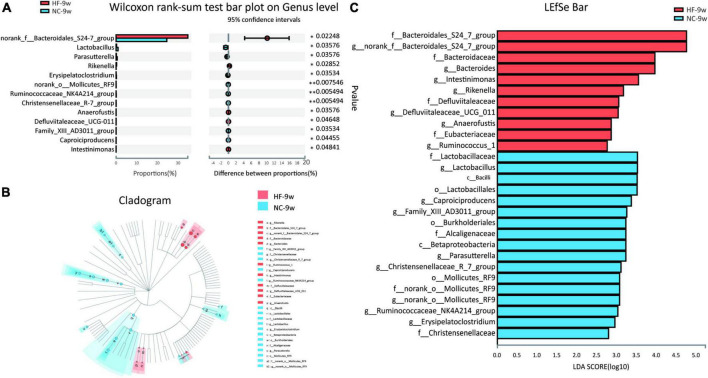
Dysbiosis of gut microbiota induced by the maternal HF diet was partly recovered in adult offspring. **(A)** Column chart of species with significant differences at the genus level. **(B,C)** LEfSe analysis of the gut microbiota from the phylum level to the genus level with LDA values of 2.0 (NC-9w, *n* = 6; HF-9w, *n* = 5). *p < 0.05, **p < 0.01 vs. NC-9w. NC, normal chow diet; HF, high-fat diet. LEfSe, linear discriminant analysis (LDA) effect size.

The Wilcoxon rank sum test was further applied to analyze differential species at phylum and genus levels in adult offspring. No significant difference was observed at the phylum level of microbial composition in adult offspring (data were not shown). At the genus level, 11 genera were found to be significantly different in adult offspring between the two groups, four genera of which were also different at weaning. These included genus *norank f Bacteroidales S24-7 group*, *Parasutterella*, *Ruminococcaceae NK4A214 group*, and *Family XIII AD3011 group*. The relative abundance of *Parasutterella* was decreased in the adult offspring of HF dams (*P* < 0.05, Figure 8A) as in the offspring of HF dams at weaning. *Parasutterella* belongs to the phylum Proteobacteria, class Betaproteobacteria, order Burkholderiales, and family Alcaligenaceae. Consistent with the offspring at weaning, the relative abundance of class Betaproteobacteria, order Burkholderiales, and family Alcaligenaceae was decreased in the adult offspring of HF dams. LEfSe revealed that the genus *Lactobacillus* from the phylum Firmicutes was the most abundant in the NC group (Figures 8B,C). The genus *norank f Bacteroidales S24-7 group* and *Bacteroides* from the phylum Bacteroidetes were enriched in the HF group (Figures 8B,C).

**FIGURE 9:**
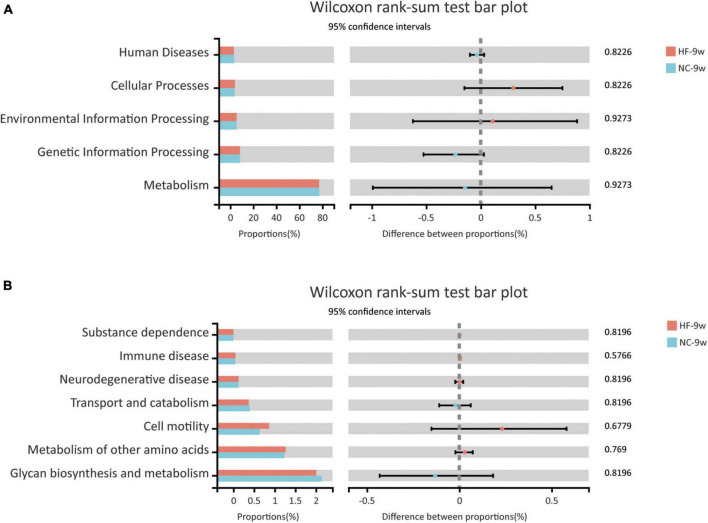
Functional predictions of bacterial communities by the KEGG pathway database in offspring at 9 weeks of age. **(A)** KEGG pathway level 1. **(B)** KEGG pathway level 2 (NC-9w, *n* = 6; HF-9w, *n* = 5). NC, normal chow diet; HF, high-fat diet.

Predicted functional metagenomic profiles based on the KEGG pathway showed that there was no difference in the metabolic function between the two groups (Figure 9).

## Discussion

Early-life adverse environmental factors are linked to the development of diabetes and obesity (1). Accumulating evidence pointed out that maternal overnutrition not only caused metabolic disturbance in dams but also determined susceptibility to obesity and diabetes in the offspring (5, 28). Growing evidence suggests an important role of dysbiosis of offspring microbiome influenced by maternal overnutrition in glycolipid disorders of offspring at weaning (6, 28, 40). However, the relationship between microbiota and metabolic disorders of offspring and whether the adverse effects caused by maternal overnutrition can be reversed by a normal diet are largely unknown. Data from our study showed that the maternal HF diet impaired glucose metabolism in dams. In line with this, a maternal HF diet negatively impacted the glycolipid metabolism and intestinal microbiota at weaning. Although dysbiosis of gut microbiota was not fully recovered by a 6-week normal diet after weaning, the disturbance of glucose and lipid was reversed in adult offspring. Not fully recovered gut microbiota may have a role in the promotion of susceptibility to obesity and diabetes in offspring when encountering metabolic stress in later life.

Our previous results demonstrated that a maternal HF diet led to higher blood glucose, glucose intolerance, and hypercholesteremia in dams during gestation (5). In the present study, the HF-fed dams exhibited glucose intolerance after lactation. These results suggested that the maternal HF diet during the perinatal period induced glucose metabolic disturbance, which will contribute to creating an adverse developmental environment for their offspring during intrauterine and early postnatal life. Consisted with the previous literature (6, 28), we found maternal HF feeding increased body weight, blood glucose, and insulin and induced glucose intolerance in male offspring at weaning. Greater fat mass and higher levels of T-CHO and LDL-C were observed in the male offspring from HF dams at weaning. Leptin and adiponectin are hormones predominately secreted from the adipose tissue (41, 42). Leptin, which is in proportion to the size of fat stores, plays a critical role in glucose homeostasis, maintaining adipose tissue mass and immune function (43, 44). Obesity-associated hyperleptinemia and leptin resistance have been related to insulin resistance, type 2 diabetes, and diabetic vascular complications (44, 45). Adiponectin, which exerts beneficial effects on insulin sensitivity and anti-inflammatory activity (46), is downregulated in settings of type 2 diabetes and obesity (47, 48). The ratio of adiponectin to leptin is a reliable biomarker for insulin resistance and diabetes (49). In the present study, the level of serum leptin was significantly increased, whereas the level of adiponectin was decreased in male offspring from HF dams at weaning. The ratio of adiponectin to leptin was dramatically decreased in the male offspring of HF dams. These results indicated that a maternal HF feeding exerted detrimental effects on the glucose and lipid homeostasis in the male offspring at weaning.

Gut microbiota performs an essential role in maintaining host glucose and lipid homeostasis (15–17). Given the intestinal microbiome of pups could be modified by a maternal diet, we investigated the impact of a maternal HF diet on the structure and diversity of gut microbiota in offspring. Consistent with previous studies (6, 40, 50), we found that the maternal HF diet led to marked changes in the gut microbiota of offspring characterized by an increased Firmicutes/Bacteroidetes ratio at the phylum level. Previous studies showed that the relative proportion of Bacteroidetes was decreased in obese people (51) and animals fed with an HF diet (52, 53). The shift in the Firmicutes/Bacteroidetes ratio at the phylum level was linked to some metabolic disorders, such as obesity and diabetes (54). The abundance of genus *norank f Bacteroidales S24-7 group* was significantly decreased by the maternal HF diet in the present study. The *norank f Bacteroidales S24-7 group* is a butyrate-producing bacterium (55, 56). It is reported that a lower level of *norank f Bacteroidales S24-7 group* was observed in HF-fed mice, db/db mice, and the offspring of HF dams (56, 57). In the present study, the butyrate level was decreased in the offspring of HF dams at weaning. Butyrate plays a critical role in protecting the gut barrier function and preventing the migration of pro-inflammatory molecules to the liver (58). Butyrate supplements reduced body weight, suppressed hepatic fat accumulation, and decreased cholesterol synthesis (28, 59–61). The underlying mechanisms are associated with increased energy expenditure and improved lipid oxidation and suppression of the expression of pro-inflammatory molecules (59–61).

The abundance of Gram-negative sulfate-reducing genus *Desulfovibrio* was significantly elevated by the maternal HF diet in offspring at weaning. The genus *Desulfovibrio* was found enriched in patients with GDM (62) and type 2 diabetes (63, 64). *Desulfovibrio* can produce LPS as an endotoxin. LPS can provoke low-grade inflammation and thus aggravates inflammation-related chronic conditions such as adiposity and insulin resistance (65). In our study, *Desulfovibrio* and Desulfovibrionaceae were enriched in HF groups at weaning and positively correlated with body weight, blood glucose levels, fat mass, and serum lipids levels. *Lachnoclostridium* is a microbial trimethylamine (TMA)-producing bacterium, which can convert choline to TMA effectively. TMA can be transformed into trimethylamine-N-oxide (TMAO) in the liver (66). TMAO was reported to be linked to the progression of obesity, diabetes, and cardiovascular diseases (67–69). An integrated metagenomic analysis of the TMA-producing bacteria in the human gut revealed that *Lachnoclostridium* was enriched in atherosclerotic patients (70). The higher abundance of *Lachnoclostridium* was negatively correlated with lower levels of acetate in circulation, which resulted in increasing visceral mass (71). In the present study, our results found that *Lachnoclostridium* was enriched in the offspring of maternal HF dams at weaning and had a positive association with FBG, fat mass, insulin, and leptin. *Blautia*, which belongs to the order Clostridiales, can transform undigested carbohydrates and proteins into acetic acid, thus regulating energy homeostasis for the human body (72). In recent investigations, a higher abundance of *Blautia* was found in type 1 diabetic children, type 2 diabetic patients, and HF-feeding mice (73–77). Meanwhile, it indicated that *Blautia* had a positive relationship with serum long-chain triglycerides (75), glucose, and HbA1c (76). In the present study, we showed that *Blautia* was enriched in offspring due to a maternal HF diet. Meanwhile, *Blautia* had a negative correlation with blood glucose, insulin, T-CHO, and LDL-C. However, the abundance of *Blautia* was also found negatively correlated with some diseases. Some studies reported that the representation of *Blautia* was lower in obese children (78) and some patients with type 2 diabetes (79). Many studies paid attention to the genus level, rather than the species or even strain levels. There may be differences of *Blautia* at the species level, and different species of *Blautia* may exert beneficial or adverse effects on human health (80).

To investigate whether the dysbiosis of gut microbiota and metabolism could be recovered by a normal diet, we delivered a normal diet to offspring after weaning. The results showed that higher body weight, glucose intolerance, and lipid dysfunction induced by a maternal HF diet were recovered by a normal diet in adult offspring. Then, we further explored the changes of the gut microbiota in offspring at 9 weeks of age. Alpha diversity suggested that the diversity of microbiota was lower in offspring of maternal HF dams. Although the structure of microbiota was still different between the two groups at 9 weeks old, beta diversity among the offspring of the four groups showed that the composition of microbiota was partly reversed by a normal diet. No difference was found in the microbiota at the phylum level. Compared with the offspring at weaning, the number of differential genera was fewer in adult offspring. Among the differential species, *Lactobacillus* was observed to decrease in the adult offspring of HF dams. *Lactobacillus* contains numbers of probiotic bacteria (77). The decrease in the number of *Lactobacillus* had been noted to increase the intestinal permeability, decrease proinflammatory cytokine, and ameliorate insulin resistance (81, 82). LEfSe suggested that the genus *Bacteroides* and *norank f Bacteroidales S24-7 group* were enriched in HF groups. A higher abundance of *Bacteroides* was observed in type 1 diabetic children (83, 84). The genus *norank f Bacteroidales S24-7 group* was enriched in the adult offspring of HF dams, which was opposite to the results at weaning. This apparent inconsistency may be complained about by potential feedback mechanisms on gut microbiota resulting from improved human physiology (81). These results indicated that the dysbiosis of gut microbiota was partly recovered by a normal diet. Predicted functional metagenomic profiles indicated that there was no difference between the two groups, and the glycolipid metabolism could be regulated by multiple tissues. That might be the reason why glucose and lipid profiles recovered when gut microbiota was not fully recovered.

In conclusion, our results showed that a maternal HF diet negatively impacted on metabolic homeostasis and gut microbiota at weaning. Glucose and lipid disturbance of offspring at an early age could be recovered by a normal diet after weaning, whereas intestinal dysbiosis of offspring at weaning could not be fully reversed by a 6-week normal diet, which might increase susceptibility to obesity and diabetes when encountering metabolic stress in later life. The relationship between the intestinal dysbiosis and metabolic disturbance may be related to gut microbiota-derived metabolites, such as SCFA, LPS, and TMAO. A normal diet after weaning might be a novel option for offspring to reverse metabolic disorders caused by maternal overnutrition.

## Data availability statement

The datasets presented in this study can be found in online repositories. The names of the repository/repositories and accession number(s) can be found in the article/Supplementary material.

## Ethics statement

The animal study was reviewed and approved by the Ethics Committee for Animal Experimentation of the Faculty of Peking University First Hospital (No. J201827).

## Author contributions

JZ and JQZ conceived and designed the experiments. JZ and LZ carried out the experiments. YG and HW analyzed the data. All authors contributed to the article and approved the submitted version.

## Footnotes

1
https://cloud.majorbio.com
