# Supplementary material for: The dynamic effects of maternal high-calorie diet on glycolipid metabolism and gut microbiota from weaning to adulthood in offspring mice
Source: Front Nutr. 2022 Jul 28;9:941969. doi: 10.3389/fnut.2022.941969 (PMC9343994; doi:10.3389/fnut.2022.941969)
Supplement: Supplementary file 2 [file Data_Sheet_1.docx]

Supplementary Material

## Supplementary Figures


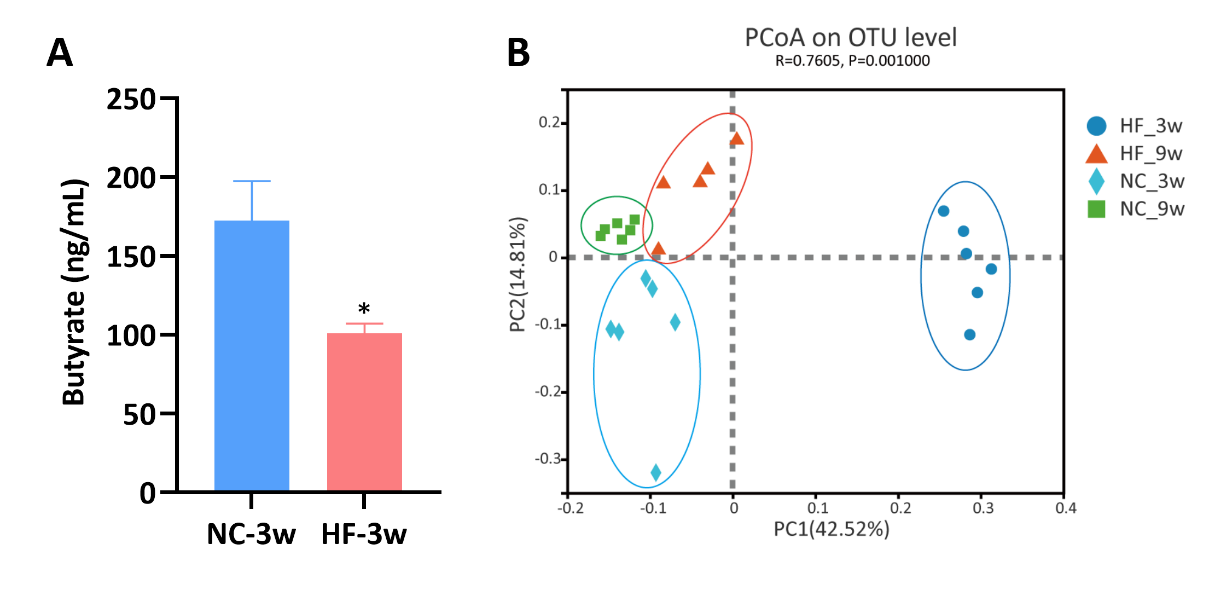


**Supplementary Figure 1.** Serum butyrate level in offspring at weaning and PCoA plots of gut communities in offspring at weaning and 9 weeks of age. **(A)** Serum Butyrate of offspring at weaning; **(B)** PCoA plots of gut communities in offspring at weaning and 9 weeks of age. (NC-3w, n=6; HF-3w, n=6; NC-9w, n=6; HF-9w, n=5). NC, normal chow diet; HF, high-fat diet.
